# A Study Over Thiol Disulfide Homeostasis in Cord Blood in Women With Gestational Diabetes 

**Published:** 2018-12

**Authors:** Lebriz Hale Aktun, Yeliz Aykanat, Özcan Erel, Salim Neşelioğlu, Oktay Olmuscelik

**Affiliations:** 1 Department of Obstetrics and Gynecology, Istanbul Medipol University, Istanbul, Turkey; 2 Department of Biochemistry, Medical School, Yıldırım Beyazıt University, Istanbul, Turkey

**Keywords:** Thiol, GDM, Cord Blood

## Abstract

**Objective:** To gain insight into the effect of gestational diabetes mellitus (GDM) on cord blood dynamic thiol/disulfide homeostasis.

**Materials and methods:** A prospective case-control study was carried out for 132 pregnant women (62 GDM, 70 controls). The cord blood samples were collected from all the participants, and the native thiol-disulfide exchanges were examined by means of an automated method enabling to measure thiol-disulfide balance. In addition to investigating shifts in thiol disulfide balance between GDM and healthy pregnancies, we sought to correlate the thiol / disulfide homeostasis parameters with other clinical and laboratory characteristics of patients with GDM and using insulin (n = 19) and on a diet only (n = 43).

**Results:** Disulfide amounts, disulfide/native thiol and disulfide/total thiol rates increased (p < 0.001) while native thiol/total thiol decreased in the cord blood of infants born to diabetic mothers (p < 0.001). Furthermore, the patient group administered with insulin and diet only was compared. Disulfide, Disulfide/Native thiol*100, Disulfide/total thiol*100, Native/total thiol*100 differ significantly according to the results (p < 0.05). Disulfide, Disulfide / native thiol * 100, Disulfide/total thiol*100, HbA1c and 75gr are higher than those in patients who do not use insulin. Only Native/total thiol*100 values are higher in patients who use insulin than those who do not.

**Conclusion:** This study suggests that the infants born to diabetic mothers are exposed to increased oxidative stress. In addition, the patients who use insulin better control their blood glucose, thus reducing the need of newborns for intensive care.

## Introduction

Gestational Diabetes Mellitus (GDM) is one of the most common metabolic disorders diagnosed during pregnancy and it refers to carbonhydrate intolerance in various levels (1, 2).

GDM screening is often performed in the week 24 to 28 during pregnancy whereas high-risk patients are recommended to be screened at the onset of pregnancy (3). 

Fetal and maternal complication risks should be minimized by following treatment and monitoring protocols in patients with GDM (4). The first-line treatment of GDM is medical diet therapy and exercise. However, insulin therapy can be administered if diet and exercise alone are inadequate.

It has been suggested that there is a well-described relation between diabetes and oxidative stress (5).

Thiols are organic compounds that contain a sulfhydryl (-SH) group composed of a sulfide bonded with a hydrogen and a carbon (6). Thiol groups are reversibly oxidized under oxidative stress in order to create disulfide bonds, a residual of two cysteine amino acids in proteins. These disulfide bonds may be reduced to thiol groups again in order to sustain the dynamic thiol-disulfide homeostasis, and there is mutual transformation between thiols (reduced state) and disulfide groups (oxidized state). This is how thiol/disulfide homeostasis is sustained (7). Total thiols contain reduced and oxidized thiols while natural thiols contain reduced thiols only (8).

Thiols contribute to substantial part of total antioxidants in body, and play a major role in defense against reactive oxygen species (9). It is a recent fact that the pathogenesis of various acute and chronic diseases offers abnormal thiol/disulfide homeostasis (8, 10). The generation of reactive oxygen species (ROS) is associated with a variety of diseases such as obesity, type II diabetes and cancer (12). Thiol measurement in serum samples provides indirect reflection of antioxidant defense (8, 10, 11-13). It has been found out that the oxidative stress increases in pregnant women with both type I diabetes and GDM caused by excessive generation of ROS and/or lack of antioxidant defenses system, and thus the imbalance in thiol redox homeostasis is associated with diabetes and complications (14, 15).

In this study, we aimed at determining the status of dynamic thiol / disulfide homeostasis as a marker of oxidative stress recently in the cord blood of GDM patients. We also investigated the correlation between thiol / disulfide homeostasis parameters and other clinical and laboratory characteristics of patients with GDM using insulin and diet only.

## Materials and methods

Upon the approval of the study ethics committee, Medipol University was projected to serve as a prospective case control study. Between January 2017 and August 2017, a total of 470 pregnant women were screened for GDM with 75 g OGTT in week 24 to 28 of pregnancy at Istanbul Medipol University Hospital's Outpatient Clinics of Gynecology and Obstetrics. Those who had spontaneous pregnancies between 18-40 years of age followed by our first antenatal visit at our hospital, those who gave birth after > 37 weeks and those who underwent other laboratory tests were included in the study. Those with multiple pregnancy, antenatal hemorrhage, grand multipara (> 4 births), a systemic disease, type 1 and type 2 diabetes, pregnancy with known pre-pregnancy obesity (BMI > 30 kg/m) or known malformation or chromosomal anomaly fetus and those with spontaneous preterm labor and those with diabetes mellitus in first-degree relatives were excluded from the study. The gestational age was calculated by measuring CRL on the first day of the last menstrual period and the first trimester USG. (10, 11). A total of 140 pregnant women (70 GDM, 70 controls) started to work. However, a total of 132 pregnant women (GDM n = 62) (control n = 70) were included in the study as a result of the exclusion of those who could not receive cord blood for technical reasons or who did not deliver at our hospital.

According to IADPSG 75GR OGTT (at least 1 pathological value is enough to diagnose) APG ≥ 92, 1.hr PG ≥ 180, 2.hr ≥ 153 (16, 17).

Those diagnosed with GDM were administered with a diet while those, who failed to be regulated by a diet, were administered with insulin therapy. Those administered with insulin therapy (n = 19) and patients administered solely with medical nutrition therapy were divided into two groups named the diet group (n = 43).

The patients on a diet were followed up for their blood glucose for 15 days. The patients, whose preprandial and pre-course blood glucose was > 95 mg/dl, 2nd-hour Pre-Prandial Blood Glucose ≥ 140 mg/dl, were administered with insulin therapy. The objectives of the therapy were in line with the recommendations set out by American Diabetes Association (ADA) (18, 19).

Immediately after the birth of the neonate, the umbilical cord was clamped and venous blood sample was collected. The samples were processed within 10 minutes after the withdrawal by centrifugation at 5000 revolutions/minute for 10 minutes, and stored at −80°C until an analysis was made.

The cord blood samples of 132 pregnant women giving a full-term birth were taken and compared to thiol/disulfide homeostasis parameters as well as demographic, obstetric and neonatal laboratory results. In addition, out of the patients diagnosed with GDM, those administered with diet only and those on insulin were compared.

***Measurement of dynamic thiol/disulfide homeostasis:*** Thiol/disulfide homeostasis was determined by a novel spectrophotometric method previously described by Erel and Neselioglu (8). 

***Data Collection Tools and Techniques:*** The data analyses were tested by means of the package program called IBM SPSS Statistics 24.0 (Statistical Package for Social Science.

Since 75 gr of pre-prandial blood glucose was not normally dispersed as suggested by the analysis, Mann Whitney U-Test was performed. The Chi Square Test was performed in order to gain insight into the relation between the variables of AF/poli, AF/olig and AF/normal, and the use of insulin. The reason why the test results include Fisher's Exact Test scores rather than Pearson Chi-Square test scores is the fact that there were scores fewer than 5 for cross table cells. ROC analysis was conducted for the patients with diabetes to find out their scores of cut-off and sensitive specificity. The results were interpreted in accordance with Youden's Index, a method usually adopted on break points calculated by 2 types of methods.

## Results

Given the similarity between gravida and birth style of a total 132 pregnant (62 with GDM and 70 control group), result findings were similar as 77.5% in GDM group and 62.9% in control group were nulliparous while 51.9% in GDM group and 58.5% in control group were vaginal birth. 30.6% of pregnant (n = 19) in GDM group used insulin and all of them were on a diet while only 7.2% of the patients in the control group were on a diet. AFI abnormality was 21% for the GDM group while the control group showed no signs of AFI abnormality (% 0). The ratios of neonates who had an apgar score of less than seven between the minutes of 1 to 5 were 4.8 % vs zero in GDM and control groups accordingly. The need of newborns for intensive care was 25.8% for the GDM group while it was 5.7% for the control group.

Table 1 shows the descriptive statistics of the patient and control groups by their laboratory clinical and anthropometric results.

The variables including disulfide, disulfide/native thiol*100, disulfide/total thiol*100, native/total thiol*100, HbA1c, 75gr preprandial blood glucose, 75gr 2nd-hour and weight at birth showed significant difference (p < 05). The scores of disulfide, disulfide/native thiol*100, disulfide/total thiol*100, HbA1c, 75gr pre-prandial blood glucose, 75gr 2nd-hour and weight at birth were higher in the patient group than the control group. The sole native/thiol*100 score was higher in the control group than the patient group. The comparison of the patient and the control group for variables such as native thiol, total thiol, age, pre-pregnancy BMI and weight gained during pregnancy showed no statistically significant difference (p > 0.05).

Table 2 compares the patient group by using and not using insulin. The variables including disulfide, disulfide/native thiol*100, disulfide/total thiol*100, native/total thiol*100, HbA1c, 75gr pre-prandial blood glucose showed significant difference (p < 0.05). The scores of disulfide, disulfide/native thiol*100, disulfide/total thiol*100, HbA1c, 75gr pre-prandial blood glucose were higher in the patients administered with insulin than those who were administered with such. 

**Table 1:** Descriptive statistics for the groups: Patient & Control Group Mann Whitney U (MWU) / T-Test Results

**Criterion**	**GDM**	**Control**	**p**	**Test**
Native thiol	343.22 ± 89.44	347.09 ± 83.55	0.770	MWU
Total thiol	388.82 ± 87.14	368.70 ± 88.10	0.060	MWU
Disulfide	22.76 ± 9.62	10.79 ± 4.61	0.000*	MWU
Disulfide/native thiol*100	7.62 ± 6.11	3.15±1.16	0.000*	MWU
Disulfide/total thiol*100	6.24 ± 3.63	2.94±1.03	0.000*	MWU
Native/total thiol*100	87.49 ± 7.24	94.12±2.06	0.000*	MWU
HbA1c	5.66 ± 0.52	5.25 ± 0.26	0.000*	MWU
75gr preprandial blood glucose	90.26 ± 3.71	86.66 ± 3.26	0.000*	T Test
75gr 1^st^hour	197.18 ± 15.57	144 ± 13.01	0.000*	MWU
75gr 2^nd^hour	171.85 ± 14.94	129.04 ± 3.74	0.000*	MWU
Age	28.11 ± 2.97	28.00 ± 302	0,749	MWU
Pre-pregnancy BMI	26.33 ± 2.44	26.90 ± 2.33	0,218	MWU
Weight gained during pregnancy	13.05 ± 1.64	13.15 ± 1.61	0,690	MWU
Weight at birth	3393.55 ± 297.99	3254.91 ± 141.27	0.005*	MWU

(Mean ± S.S) Mann Whitney U (MWU) /T test

KS: Kolmogorov-Smirnov Test; SW: Shapiro-Wilk Test

The variable 75gr PBG was dispersed normally as it was p = ,137 > ,05 according to the normality test (Table 1). The variable 75 gr. preprandial blood glucose was analyzed by means of parametric tests while the other was analyzed by means of non-parametric tests.

* Statistically significant (0.05)

**Table 2:** Whitney U (MWU) / T Test Results By the Use of Insulin

**Criterion**	**Not on insulin**	**On insulin**	**p**	**Test**
Native thiol	335.96 ± 97.6	362.46 ± 61.33	0.167	MWU
Total thiol	387.56 ± 95.10	392.15 ± 63.73	0,636	MWU
Disulfide	25.75 ± 9.57	14.84 ± 3.02	0.000*	MWU
Disulfide/native thiol*100	8.93 ± 6.71	4.15 ± 0.80	0.000*	MWU
Disulfide/total thiol*100	7.16 ± 3.88	3.82 ± 0.69	0.000*	MWU
Native/total thiol*100	85.65 ± 7.71	92.35 ± 1.37	0.000*	MWU
HbA1c	5.82 ± 0.52	5.25 ± 0.22	0.001*	MWU
Age	27.96 ± 3.06	28.53 ± 2.76	0.352	MWU
Pre-pregnancy BMI	26.38 ± 2.40	26.21 ± 2.61	0,893	MWU
Weight gained during pregnancy	12.91 ± 1.61	13.41 ± 1.73	0.329	MWU
Weight at birth	3398.89 ± 298.05	3379.41±306.52	0.570	MWU

* Statistically significant (0.05)

The sole native/thiol*100 score is higher in patients on insulin therapy than those who are not. The comparison of the patients using insulin and those not using insulin for variables such as native thiol, total thiol, 75 gr. OGTT, age, pre-pregnancy BMI and weight gained during pregnancy showed no statistically significant difference (p > 0.05).

When the apgar scores are taken into account, the comparison of a total of 62 patients including 3 patients with the apgar score 7 < and 59 patients with the apgar score ≥ 7. The variables including native thiol, total thiol, disulfide, disulfide/native thiol*100, disulfide/total thiol*100, native/total thiol*100, HbA1c, 75gr 2nd-hour, weight gained during pregnancy and weight at birth showed significant difference (p < 0.05). Native thiol, total thiol and native/total thiol*100 scores were lower for the patients with the score 7 < than those with the apgar score 7 ≥. The scores of disulfide, disulfide/native thiol*100, disulfide/total thiol*100, HbA1c, 75gr 2^nd^-hour and weight gained during pregnancy were higher for the patients with apgar score 7 < than those with the Apgar score 7 ≥. The comparison of the patients with the apgar score 7 < and 7 ≥ showed no statistically significant difference in 75gr preprandial blood glucose, age and pre-pregnancy BMI (p > 0.05).

Table 3 shows the sensitivity analyses made for the patients with and without diabetes. The results were interpreted in accordance with Youden's Index, a method usually adopted on break points calculated by 2 types of methods. According to the results, the ROC analyses for disulfide, disulfide/native thiol, disulfide/total thiol and native thiol/total thiol ratios were significant (p < 0.05). The best break point for disulfide was 100.00% sensitivity and 4.44% specificity and 11.15 μmol/L while AUC score turned out to be 0.119 (95%Cl: 0.034-0.204). The best breaking point for disulfide/native thiol ratio was 100.000% sensitivity and 0.00 specificity and 1.71 μmol/L while the AUC score turned out to be 0.0142 (95%Cl:0.053-0.232). The best breaking point for disulfide/native thiol ratio was 100.00% sensitivity and 0.00% specificity and 1.57 μmol/L while the AUC score turned out to be 0.142 (95%Cl:0.052-0.232). The best break point for native thiol/total thiol ratio was 100.000% sensitivity and 71.11% specificity and 89.86 μmol/L while the AUC score turned out to be 0.867 (95%Cl:0.781-0.954).

**Table 3:** ROC Tests For Patients With Diabetes Cut-off Levels, Specifity and Sensitivity Test Results

**Cutoff Selection Criterion**	**Youden's index**			**Pathagoras' theorem**			**Auc (95%Cl)**	**P**
**Evaluation Criteria**	**Cut off**	**Spe %**	**Sen %**	**Cut off**	**Spe %**	**Sen %**		
Native thiol	288.65	94.12	31.11	338.80	64.71	55.56	0.614 (0.467-0.762)	0.167
Disulfide	11.15	100.00	4.44	11.15	100.00	4.44	0119 (0034-0204)	0.000*
Total thiol	364.10	70.59	44.44	368.85	64.71	48.89	0,539 (0,384-0,695)	0,636
Disulfide/native thiol ratio	1.71	100.00	0.00	4.01	64.71	13.33	0,142 (0,053-0,232)	0.000*
Disulfide/total thiol ratio	1.57	100.00	0.00	3.71	64.71	13.33	0,142 (0,052-0,232)	0.000*
Native thiol/total thiol	89.86	100.00	71.11	90.46	94.12	75.56	0,867 (0,781-0,954)	0.000*

* Statistically significant (0.05) AUC; area under the curve, CI; Confidence Interval

## Discussion

The results show that disulfide, disulfide/native thiol*100 and disulfide/total thiol*100 were higher in the GDM group than the control group. The sole native/thiol*100 score was higher in the control group than the patient group.

Our study suggests that the thiol/disulfide homeostasis in cord blood of mothers with GDM shifts in favor of disulfide, thus causing an increase in thiol oxidation. All the past studies over the balance between the oxidative stress in cord blood and antioxidant defense systems reported an increase in oxidative stress markers (E.g.: MDA and xanthine oxydase) and a significant decrease in antioxidant enzymes superoxide dismutase, catalase, glutathione peroxidase) (20, 21). Ates et al. reported in a study over dynamic thiol/disulfide homeostasis that the native thiol levels were significantly low in pre-diabetic patients (22). This is in compliance with the results of this study as we reduced the native thiol levels in cord blood of diabetic mothers and the native/total thiol ratio. Harper et al. showed in a study that the excess weight gained by patients with GDM has a correlation with macrosomia and negative pregnancy outcomes for newborns (23). Our study showed no significant difference between the patient and the control group in pre-pregnancy BMI, weight gained during pregnancy and weight at birth. Despite this, those with the apgar score < 7 had significantly higher scores of weight gained during pregnancy and weight at birth. We found out that as the patients with higher scores of BMI were excluded, the weight gained during pregnancy and weight at birth were in correlation with the low apgar score for the patients with GDM. This may be associated with the limited number of patients involved in the study. Ozler et al. reported similar results in a study and argued that the thiol/disulfide homeostasis for patients with GDM shifted in favor of homeostasis, thus causing an increase in thiol oxidation (24, 25).

In addition, it was found out that the increased disulfide in cord blood and the decreased native/total thiol rate was significantly in correlation with the low apgar score. This shows that the increased thiol oxidation may be correlated with negative perinatal outcomes in GDM (increased need for NICU). As untreated GDM is risky for perinatal morbidity, it is crucial to make early estimations for potentially negative outcomes of GDM to prevent potential complications. Our study shows a comparison of patients administered and not administered with insulin. The variables including disulfide, disulfide/native thiol*100, Disulfide/total thiol*100 and native/total thiol*100 showed significant difference (p < 0.05). The scores of disulfide, Disulfide/native thiol*100, Disulfide/total thiol*100, HbA1c, 75gr pre-prandial blood glucose were higher in the patients administered with insulin than those who were administered with such. The sole native/thiol*100 score is higher in patients on insulin therapy than those who are not. Our study is in agreement with the results of the previous studies indicating that the probability of need for insulin can be projected based on abnormal OGTT values (26, 27).

## Conclusion

In conclusion, we suggested that the offspring born to diabetic mothers are exposed to increased oxidative stress. In this study, we found out that 75gr OGTT were predictive for the future need for insulin therapy in achieving optimal blood glucose regulation in 24–28 weeks of pregnancy. In addition, the patients who use insulin better control their blood sugar, thus reducing the need of newborns for intensive care.

The main restriction on our study was the relatively few number of patients that might limit the applicability of the results on the general population. Therefore, it is necessary to carry out further studies with larger cohorts to confirm the results of this study.
